# Antiretroviral therapy uptake and coverage in four HIV community cohort studies in sub-Saharan Africa

**DOI:** 10.1111/j.1365-3156.2011.02925.x

**Published:** 2012-07-30

**Authors:** Alison Wringe, Sian Floyd, Patrick Kazooba, Phyllis Mushati, Kathy Baisley, Mark Urassa, Anna Molesworth, Christina Schumacher, Jim Todd, Basia Zaba

**Affiliations:** 1 London School of Hygiene and Tropical MedicineLondon, UK; 2 Medical Research CouncilEntebbe, Uganda; 3 Biomedical Research and Training InstituteHarare, Zimbabwe; 4 National Institute of Medical ResearchMwanza, Tanzania; 5 Imperial CollegeLondon, UK

**Keywords:** antiretroviral therapy, access, coverage, sub-Saharan Africa

## Abstract

**Objective:**

To compare socio-demographic patterns in access to antiretroviral therapy (ART) across four community HIV cohort studies in Africa.

**Methods:**

Data on voluntary counselling and testing and ART use among HIV-infected persons were analysed from Karonga (Malawi), Kisesa (Tanzania), Masaka (Uganda) and Manicaland (Zimbabwe), where free ART provision started between 2004 and 2007. ART coverage was compared across sites by calculating the proportion on ART among those estimated to need treatment, by age, sex and educational attainment. Logistic regression was used to identify socio-demographic characteristics associated with undergoing eligibility screening at an ART clinic within 2 years of being diagnosed with HIV, for three sites with information on diagnosis and screening dates.

**Results:**

Among adults known to be HIV-infected from serological surveys, the proportion who knew their HIV status was 93% in Karonga, 37% in Kisesa, 46% in Masaka and 25% in Manicaland. Estimated ART coverage was highest in Masaka (68%) and lowest in Kisesa (2%). The proportion of HIV-diagnosed persons who were screened for ART eligibility within 2 years of diagnosis ranged from 14% in Kisesa to 84% in Masaka, with the probability of screening uptake increasing with age at diagnosis in all sites.

**Conclusions:**

Higher HIV testing rates among HIV-infected persons in the community do not necessarily correspond with higher uptake of ART, nor more equitable treatment coverage among those in need of treatment. In all sites, young adults tend to be disadvantaged in terms of accessing and initiating ART, even after accounting for their less urgent need.

## Introduction

Various approaches have been taken to scaling up HIV testing and treatment services in sub-Saharan Africa, depending on human resource availability, funding mechanisms, national policy and health infrastructure. In particular, alternative models to traditional voluntary counselling and testing (VCT) services, including mobile, door-to-door or workplace VCT services, and opt-out HIV testing in health facilities, are increasingly being promoted to improve low HIV diagnosis rates (Matovu & Makumbi 2007; WHO 2008). While these strategies can increase the number of persons who learn their status (Menzies *et al.* 2009), they may also increase the proportion of HIV-infected persons who know their positive status but fail to enrol in HIV treatment programmes.

A recent systematic review of pre-antiretroviral therapy (ART) retention in care in Africa showed that the median rate of attrition between receiving a HIV diagnosis and subsequently enrolling at a HIV treatment clinic was 41% (range: 12–65%), while the median attrition rate from staging to ART eligibility was 54% (5–69%) and from ART eligibility to treatment initiation was 32% (16–86%) (Rosen & Fox 2011). However, none of the included studies provided a complete picture in terms of dropout at every stage of the process of accessing ART – from the time of diagnosis right through to ART initiation – or related these findings to estimated ART coverage in the population. Such an analysis would require information on the estimated proportion of HIV-infected persons who need HIV treatment, which is itself a function of the stage of the epidemic and the age distribution of HIV incidence over time (Zaba *et al.* 2012).

Community-level HIV cohort studies provide a unique opportunity to track progress in coverage with HIV testing and treatment services in relation to need for treatment, as HIV programmes are rolled out. The availability of age-specific HIV prevalence data and mortality rates enables estimates of ART need to be made by characteristics including age, sex and area of residence (Zaba *et al.* 2012). Data on HIV service use including HIV testing, referral and ART clinic attendance can be anonymously linked to data on the total community population, so that the proportion of all HIV-infected persons who have accessed each service can be estimated at a given point in time. Cross-country comparisons can shed light on the extent to which different strategies for providing HIV testing and treatment have resulted in higher HIV testing and treatment coverage, or greater equity in terms of ART coverage, providing information that is important for informing current HIV testing and treatment policies. This study compares HIV testing and treatment uptake in four cohort studies in Malawi, Tanzania, Zimbabwe and Uganda.

## Methods

### Participating sites and study settings

Four HIV cohort studies participating in the ALPHA network contributed data for this analysis: Karonga (Malawi), Kisesa (Tanzania), Masaka (Uganda) and Manicaland (Zimbabwe). Sites were selected if they collected data on participants’ use of HIV testing services and ART initiation, and preferably referral to HIV treatment clinics. These data were collected either from (i) attendance records at local HIV testing and HIV treatment clinics that could be anonymously linked to the data collected during the serological survey rounds or (ii) from self-reported information collected from questionnaires administered during the serological surveys.

Table 1 summarises the data sources used and the dates that HIV services were introduced for each site in the analysis. A description of the study setting, data collection methods and HIV service availability for each site is also provided below. More detailed descriptions for each study setting and the construction of each dataset are provided in previous publications (Cremin *et al.* 2009; Floyd *et al.* 2010) and in site-specific analyses of VCT and ART uptake that are presented elsewhere in this supplement (Kazooba *et al.* 2012).

**Table 1:** Characteristics of each study site

Site	Karonga	Kisesa	Masaka	Manicaland
HIV service availability				
Date of VCT introduction (excluding serosurveys)*	2005	2005	2004	2000
First serosurvey round with VCT	2007–2008	2003–2004	1989	2000
Date of free ART introduction in the study area	June 2005	March 2005	January 2004	January 2007†
Criteria for ART initiation	CD4 < 200 (250 Karonga), or WHO stage 4/stage 3‡; CD4 < 250 for pregnant women			
Data sources				
Data source for VCT use and date	Reported, home-based	Reported, clinic	Reported, clinic	Reported
Data source for referral to ART clinic and date	Reported, clinic	Clinic	N/A	N/A
Data source for ART clinic registration and date	Clinic	Clinic	N/A	N/A
Data source for ART screening and date	Clinic	Clinic	Clinic	N/A
Data source for ART eligibility and date	Clinic	Clinic	Clinic (CD4 only)	N/A
Data source for ART initiation and date	Reported, clinic	Clinic	Clinic	Reported, no date
Cross-sectional analysis				
Year of survey	2007–2008	2006–2007	2008	2006–2008
HIV prevalence at survey	6.3% (M), 8.5% (F)	5.1% (M), 6.7% (F)	5.9% (M), 8.9% (F)	12.5% (M), 18.8% (F)
Number of HIV+ eligible for survey	837	676	575	2057
Prospective analysis				
Residence eligibility for inclusion of HIV+, ART-naive persons in the analysis	Resident at 2007–2008 survey; interviewed after January 2008	Resident at 2006–2007 survey	Resident at 2005 or 2006 survey	N/A
Period of diagnosis	January 2008–October 2008	2005–2007	2004–2006	N/A
Period of screening	January 2008–June 2010	2005–2009	2004–2008	N/A
Persons included in the analysis	473	676	636	N/A

ART, antiretroviral therapy; VCT, voluntary counselling and testing.

* Since the availability of ART.

† ln Manicaland, ART was introduced in the study area in 2005 with very limited availability and with scale-up occurring gradually from 2007onwards.

‡ Specific stage 3 conditions in Masaka, Kisesa and Manicaland.

#### Karonga

Karonga district is a rural area in northern Malawi, where demographic surveillance surveys (DSS) have been conducted among a sub-population of 33 500 individuals since 2002–2004, collecting monthly information on births and deaths, and migration information each year. Serological surveillance has been conducted annually since September 2007 among all individuals aged 15 years or over residing in the DSS area. HIV testing, with pre- and post-test counselling, is offered at participants’ homes, using rapid tests, with referrals made to the nearest ART clinic for those with an HIV-positive diagnosis. In addition, a detailed questionnaire is administered covering prior VCT use, referral to HIV clinics and ART use.

Antiretroviral therapy was first available to the study population at a district clinic in June 2005. By the time of the first serological surveillance round, additional health facilities were providing ART, including one within the DSS area. Data from patient records at the ART clinic in the DSS area have been anonymously linked to cohort records since January 2008.

#### Kisesa

The Kisesa cohort study comprises five rural villages and a trading centre in north-west Tanzania. Serological surveys are conducted every 3 years, with demographic surveillance carried out separately during biannual household visits among approximately 29 000 individuals (Mwaluko *et al.* 2003). During serological surveys, HIV testing was conducted for research purposes without results disclosure among consenting adults, and a detailed questionnaire was administered covering health service use and sexual behaviour.

Since the 2003–2004 serological survey, separate, temporary VCT services were offered to participants in each village. In addition, a government-run VCT clinic was available at the local health centre within the surveillance area since 2005, with free ART available at a referral hospital 20 km away. Referrals to the ART clinic were made from the survey VCT and the health centre VCT services since the ART programme started, with data from referral forms and the ART clinic linked to the VCT data using numerical identifiers (Nsigaye *et al.* 2009). The VCT data have been anonymously linked to the cohort data since 2005.

#### Masaka

The Masaka cohort study is located in rural south-west Uganda. An annual household survey has been conducted since 1989, with all residents in the study area eligible for inclusion (Mbulaiteye *et al.* 2002). Demographic and HIV surveillance data are collected during annual house-to-house rounds. Survey participants who want to learn their status are referred to local VCT centres, where they are informed of their result, following counselling.

In addition, one-third of the HIV-infected adults who participated in the first survey in 1990 were selected at random to participate in a ‘clinical cohort’ (Morgan *et al.* 1997). The clinical cohort now includes all HIV seroconverters from subsequent rounds, together with age-matched HIV-negative controls. Participants attend medical follow-up visits at the study clinic every 3 months, with ART provision available for eligible and consenting participants since January 2004. The clinic also provides HIV treatment to others living in the study area and is the sole ART provider in the area.

#### Manicaland

The Manicaland cohort study in Zimbabwe is a population-based open cohort of men and women aged 15–54 in 12 communities representing four socio-economic strata – (i) small towns; (ii) large-scale tea, coffee and forestry estates; (iii) roadside trading settlements; and (iv) subsistence farming areas. Prior to each survey, eligible participants were identified through a household census. Detailed information on demographics, sexual behaviour and health service use was collected through face-to-face interviews every 2–3 years between 1998 and 2008 (Gregson *et al.* 2006; Cremin *et al.* 2009). Anonymous HIV testing for research purposes was carried out at the time of the interview. All participants were offered free VCT provided by trained nurse counsellors from the study’s mobile VCT clinic, and infected individuals were referred to local clinics or hospitals for ART. Information on VCT service use was collected during each survey, with questions on ART use added in the 2003–2005 round.

### Statistical methods

Two analyses were undertaken using Stata 11 (StataCorp, College Station, TX, USA). The first, a cross-sectional analysis, describes the distribution of the HIV-infected adult population in each site in terms of HIV and ART services accessed at the time of a recent serological survey. The number of persons who had initiated ART was compared to the total number of HIV-infected persons, and the number estimated to be in need of treatment at the time of the survey, using the methods described below.

The second analysis prospectively assesses the delays between being diagnosed with HIV (defined as having a positive HIV test and receiving the results) and subsequently being screened at an HIV treatment clinic. This analysis includes the three sites (Karonga, Kisesa and Masaka) that had information on diagnosis and screening dates, as well as age and education level at time of diagnosis. The time-period and number of participants in the denominator for each of the two analyses, by site, are shown in Table 1.

#### HIV service coverage: a cross-sectional analysis

For the cross-sectional analysis, each site provided data from a recent serological surveillance round for everyone in the DSS population who was age-eligible for the selected serological survey. The dataset from each site included at least HIV status, HIV testing with receipt of results, initiation of ART, date of birth and sex. Three sites (Karonga, Kisesa and Masaka) provided additional information on the intermediary steps between HIV diagnosis and ART initiation, including whether a person was referred for HIV treatment, and/or whether they had registered at a HIV clinic and/or whether they had completed ART eligibility screening (Table 1).

Participants from Kisesa and Masaka were included in the analysis if they were *eligible to attend* the selected serological surveillance round and had tested HIV-positive either at the selected round, or at any previous round attended, regardless of whether the participant was aware of their HIV status. This inclusion criterion maximised the baseline number of HIV-infected persons in the population, reducing the risk of under- or overestimating HIV service coverage as a result of participation bias. Participants for Manicaland were included if they *attended* the 2006–2008 survey and had tested HIV-positive either at that round or at any previous serological surveillance round attended, as ART use was self-reported, and could thus only be obtained among those attending the 2006–2008 round. Participants for Karonga were included in the analysis if they tested HIV-positive in the 2007–2008 round, as this was the first serological survey covering the entire DSS population.

For each of the four participating sites, the following proportions were calculated among HIV-infected adults aged 15 years and older, for the available data: (i) learned their HIV status, (ii) registered at a HIV treatment clinic, (iii) completed ART eligibility screening; (iv) been eligible to initiate ART and (v) initiated ART. Cross-tabulations and bar graphs were used to compare the distribution of the use of HIV and ART services among the HIV-infected population, by age and sex across the four sites.

Age- and sex-specific estimates of ART coverage were calculated for each site by comparing the estimated number of persons in need of treatment with the number currently on ART, for the chosen survey year. The proportion of adults estimated to need therapy was determined using the methods that have been described in detail by Zaba *et al.* (2012) in this supplement. In brief, age-specific mortality among all HIV-infected adults was described using a Weibull model, fitted to mortality patterns observed in the ALPHA network sites prior to ART availability, using time-to-event parametric regression on age at death among the HIV infected (Zaba *et al.* 2007). The regression model can be summarised as:


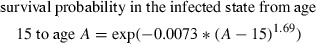


This fitted model life table allows the calculation of the conditional probability of survival for infected persons from any age for a fixed number of years in the absence of treatment. At the start of an ART programme, the proportion of infected individuals needing ART at each age was estimated as their probability of dying in the next 3 years, on the assumption that HIV-infected individuals reach a CD4 of 200 approximately 3 years prior to death in the absence of ART (Wandel *et al.* 2008). For each subsequent year of the programme, the estimated proportion needing ART was augmented by the proportion expected to die 1 year later. These predicted probabilities were then summed together to obtain an estimate of the proportion of HIV-infected adults in need of ART, among those included in the analysis.

#### Delays between receipt of a HIV diagnosis and ART screening: a prospective analysis

The prospective analysis assessed the delays between a HIV diagnosis and ART screening in three sites (Karonga, Kisesa and Masaka) that had data on HIV diagnosis date and ART eligibility screening date, enabling the delay between these two events to be calculated. Additional details on the methods for determining dates of diagnosis and screening have been described elsewhere (Floyd *et al.* 2010; Kazooba *et al.* 2012).

For Karonga, the analysis was restricted to HIV-infected participants reporting that they were ART-naive and who were interviewed after the 22 January 2008, to ensure that screening rates were not underestimated because data on ART screening from the local ART clinic were only available from the end of January 2008 up to mid-2010. Participants from Kisesa were included in the analysis if they were eligible to attend the most recent serological surveillance round in 2006–2007 and if they had tested HIV-positive in this, or any previous serological survey. Data on HIV diagnoses covered the period 2005–2007, and clinic data on ART screening covered the period from 2005 to 2009. For Masaka, the analysis was restricted to HIV-infected participants who were resident at any point during the 2005 or 2006 serological surveys and who were diagnosed by December 2006. This restriction was made because data on ART screening were only available up to the end of 2008.

The distribution of the delay between diagnosis and screening was compared across sites, by sex and age at diagnosis. Chi-square tests were conducted to identify socio-demographic characteristics associated with undergoing ART eligibility screening within 2 years of a HIV diagnosis, with stratum-specific odds ratios examined to check for possible interaction. Logistic regression was used to build multivariable models for identifying the socio-demographic factors that were independently associated with ART screening within 2 years of a HIV diagnosis.

### Ethical approval

Each study site received ethical approval from a local regulatory authority. The ALPHA network data sharing agreement covered data sharing between sites.

## Results

### Characteristics of participants

The total number of HIV-infected adults in the analysis for each site is shown in Table 2, by sex, age group and education. Manicaland had the highest number of HIV-infected individuals (*n* = 2057), while Masaka had the fewest (*n* = 575), reflecting differences in study population size and HIV prevalence. The sex and age distribution of HIV-infected persons was similar across all settings, although, in Karonga, they were slightly older than in the other sites. The proportion of HIV-infected adults with no schooling ranged from 3% in Karonga and Manicaland to 30% in Kisesa.

**Table 2:** Socio-demographic characteristics of HIV-infected adults in the cross-sectional analysis, by site

	Site			
	
	Karonga *n* (%)	Kisesa *n* (%)	Masaka *n* (%)	Manicaland *n* (%)
All HIV-infected	837 (100)	686 (100)	575 (100)	2057 (100)
Sex				
Men	308 (37)	250 (36)	214 (37)	639 (31)
Women	529 (63)	436 (64)	361 (63)	1418 (69)
Age				
15–24	73 (9)	128 (19)	79 (14)	283 (14)
25–34	297 (35)	259 (38)	188 (33)	836 (41)
35–44	255 (30)	168 (24)	186 (32)	577 (28)
45+	212 (25)	131 (19)	122 (21)	360 (18)
Education*				
None	26 (3)	208 (30)	38 (7)	66 (3)
Primary 1–4	55 (7)	83 (12)	97 (17)	137 (7)
Primary 5–7†	457 (55)	351 (51)	244 (42)	611 (30)
Secondary or more	269 (32)	34 (5)	132 (23)	1236 (60)

* 4% missing data in Karonga and 11% missing data in Masaka.

† 8 years of primary school education in Malawi.

### HIV service coverage: a cross-sectional analysis

**Figure 1:**
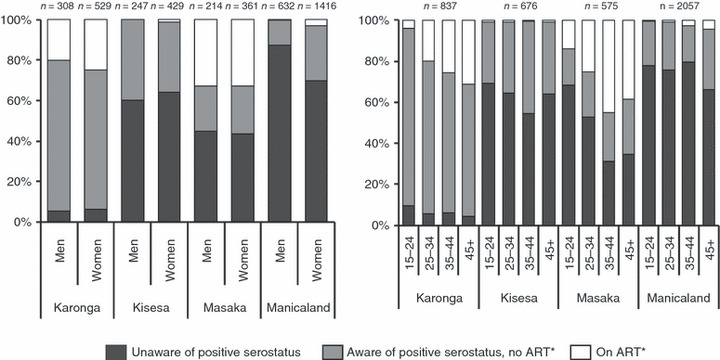
HIV service coverage among HIV-infected persons at each site, by sex (left-hand panel) and age at the selected survey round (right-hand panel). *ART data derived from self-report for Manicaland; clinic data for Kisesa and Masaka; and combined self-report and clinic data for Karonga.

HIV service coverage among HIV-infected persons by the selected survey round varied widely across sites (Table 3, Figure 1). In Karonga, 95% of infected adults had learned their HIV-positive status since the start of the national ART programme, compared with 56% in Masaka, 37% in Kisesa and 25% in Manicaland. The overall proportion of HIV-infected persons on ART was highest in Masaka (33%), followed by Karonga (23%) and was substantially lower in Manicaland (2%) and Kisesa (1%).

**Table 3:** HIV service coverage among HIV-infected persons by site: a cross-sectional analysis

	Site			
	
HIV service access	Karonga *n* (%)	Kisesa *n* (%)	Masaka *n* (%)	Manicaland *n* (%)
All HIV-infected	837 (100)	676 (100)	575 (100)	2057 (100)
Knows status	788 (94)	251 (37)	322 (56)	524 (25)
Referred	NA	84 (12)	NA	NA
Registered at HIV clinic	NA	10 (1)	NA	NA
Completed ART screening	209 (25)	10 (1)	255 (44)	NA
Ever eligible for ART*	202 (24)	6 (1)	191 (33)	NA
Currently on ART†	194 (23)	5 (1)	189 (33)	40 (2)

ART, antiretroviral therapy.

* According to screening results.

† According to self-report for Manicaland, clinic data only for Kisesa & masaka and combined self-report and clinic data for Karonga.

Among the three sites with data on enrolment into ART programmes, the proportion of HIV-diagnosed persons who had been screened for ART eligibility varied considerably between sites, being highest in Masaka (255/322; 79%) and lowest in Kisesa (10/251; 4%). More than 90% of those ever eligible to initiate ART had started treatment in all three sites.

Within each site, there was little difference in the proportions of men and women who had been diagnosed with HIV or initiated ART, with the exception of Manicaland where HIV testing uptake was lower among men (Figure 1). The proportions diagnosed with HIV, and on ART varied by age at diagnosis in all sites, with those in the younger age groups being the least likely to know their positive HIV status and those in the older age groups being most likely to have initiated ART (Figure 1).

Antiretroviral therapy coverage among those estimated to need treatment at the selected survey rounds ranged from 2% in Kisesa to 68% in Masaka (Table 4). In all sites, estimated ART coverage was higher among women than men and was generally lowest among 15- to 24-year-olds. Estimated ART coverage was lower among those with least education in all sites, except Masaka.

**Table 4:** Antiretroviral therapy (ART) coverage

Site	Variable	Category	HIV-positive	Estimated ART need	On ART*	Estimated coverage
Karonga		Total	837	334	194	(58)
					
	Sex	Male	308	130	62	(48)
		Female	529	204	132	(65)
	Age at serosurvey	15–24	73	12	3	(25)
		25–34	297	74	60	(81)
		35–44	255	81	65	(80)
		45+	212	84	66	(79)
	Education level	None	26	13	6	(46)
		Primary 1–4	55	24	10	(41)
		Primary 5–7	457	185	113	(61)
		Secondary +	269	101	60	(59)
Kisesa		Total	676	212	5	(2)
					
	Sex	Male	247	82	0	(–)
		Female	429	131	5	(4)
	Age at serosurvey	15–24	127	18	1	(6)
		25–34	254	51	2	(4)
		35–44	165	41	1	(2)
		45+	130	29	1	(3)
	Education level	None	208	72	1	(1)
		Primary 1–4	83	27	1	(4)
		Primary 5–7	351	105	2	(2)
		Secondary +	34	9	1	(11)
Masaka		Total	575	279	189	(68)
					
	Sex	Male	214	111	70	(63)
		Female	361	168	119	(71)
	Age at serosurvey	15–24	79	20	11	(54)
		25–34	188	81	47	(58)
		35–44	186	100	84	(84)
		45+	122	79	47	(60)
	Education level	None	38	21	14	(67)
		Primary 1–4	97	50	38	(76)
		Primary 5–7	244	116	73	(63)
		Secondary +	132	63	42	(66)
Manicaland		Total	2057	520	40	(8)
					
	Sex	Male	639	164	1	(1)
		Female	1418	356	39	(11)
	Age at serosurvey	15–24	283	38	2	(5)
		25–34	836	186	8	(4)
		35–44	577	169	15	(9)
		45+	360	128	15	(12)
	Education level	None	66	21	0	(–)
		Primary 1–4	137	42	4	(10)
		Primary 5–7	611	169	18	(11)
		Secondary +	1236	287	18	(6)

* Determined through self-report for Manicaland, clinic data for Masaka & Kisesa, and combined self-report and clinic data for Karonga.

### Delays between a HIV diagnosis and ART screening: prospective analysis

The proportion of diagnosed persons who were screened for ART eligibility within 2 years of their HIV diagnosis was highest in Masaka (248/295; 84%) and lowest in Kisesa (36/251; 14%). The time from diagnosis to screening was also shortest in Masaka, with 95% (236/248) of screened patients being seen within 12 months of diagnosis, compared with 86% (31/36) in Kisesa and 77% (127/164) in Karonga.

**Figure 2:**
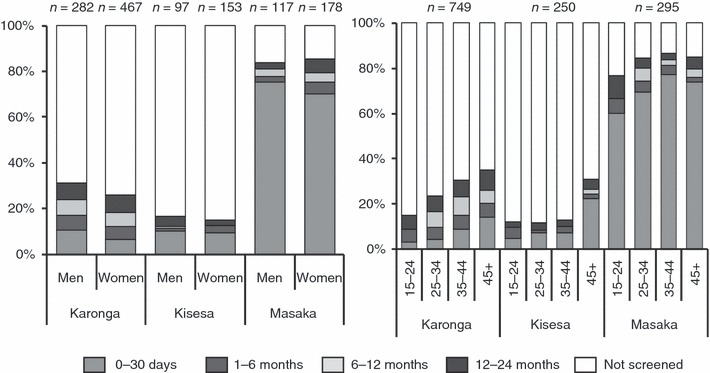
Delays between HIV diagnosis and first antiretroviral therapy screening among diagnosed adults, by site and sex (left panel) and age at diagnosis (right panel).

In all sites, the delay between a HIV diagnosis and ART screening tended to be slightly shorter among men than women (Figure 2). In Kisesa and Karonga, delays in being screened for ART following a HIV diagnosis tended to be shorter with increasing age, with a similar pattern observed in Masaka, except for the oldest age group (Figure 2).

Table 5 shows the crude and adjusted odds ratios of ART screening within 2 years of HIV diagnosis by site. In Karonga and Kisesa, age at diagnosis was associated with ART screening in the crude analysis, with evidence of higher odds of screening with older age groups (OR for trend = 1.78, *P* < 0.001 and OR for trend = 1.55, *P* < 0.017, respectively; chi-squared test for trend). In Karonga only, the odds of screening were lowest among those with secondary education, although there was only weak evidence of an overall association with education (*P* = 0.10). In Masaka, no associations were observed between the socio-demographic characteristics and ART screening in the crude analysis.

**Table 5:** Factors associated with antiretroviral therapy (ART) screening within 2 years of a HIV diagnosis

Site	Variable	Category	Had VCT *N*	Screened for ART* *n* (%)	Crude odds ratio	95% CI	Adjusted odds ratio†	95% CI
Karonga		Total	432	162 (38)				
							
	Sex	Male	170	69 (41)	1.24	(0.83–1.85)	1.03	(0.67–1.60)
		Female	262	93 (35)	1.00		1.00	
	Age at diagnosis	15–24	40	6 (15)	0.44	(0.17–1.14)	0.46	(0.18–1.18)
		25–34	169	48 (28)	1.00		1.00	
		35–44	128	56 (44)	1.96	(0.20–3.20)	1.86	(1.13–3.05)
		45+	95	52 (55)	3.04	(1.77–5.25)	3.09	(1.76–5.42)
	Education*	None/Primary 1–4	45	15 (33)	0.70	(0.36–1.37)	0.52	(0.25–1.05)
		Primary 5–8	237	99 (42)	1.00		1.00	
		Secondary+	136	42 (31)	0.79	(0.40–0.98)	0.70	(0.44–1.13)
	Residence	Rural	240	89 (37)	1.00		1.00	
		Roadside	192	73 (38)	1.04	(0.70–1.54)	0.93	(0.61–1.42)
Kisesa		Total	251	36 (14)				
							
	Sex	Male	98	14 (14)	0.99	(0.48–2.05)	0.85	(0.32–2.22)
		Female	153	22 (14)	1.00		1.00	
	Age at diagnosis	15–24	41	5 (12)	1.18	(0.38–3.72)	1.02	(0.29–3.58)
		25–34	95	10 (11)	1.00		1.00	
		35–44	69	7 (10)	0.96	(0.35–2.67)	0.59	(0.17–2.07)
		45+	46	14 (30)	3.72	(1.45–9.52)	2.57	(0.83–7.95)
	Education*	None	59	10 (17)	1.25	(0.55–2.84)	0.99	(0.36–2.72)
		Primary 1–4	35	4 (11)	0.79	(0.25–2.47)	0.40	(0.08–1.93)
		Primary 5–7 or more	157	22 (14)	1.00		1.00	
	Residence	Rural	92	7 (8)	1.00		1.00	
		Roadside	70	10 (14)	2.02	(0.72–5.67)	1.78	(0.63–5.08)
		Trading centre	77	8 (10)	1.41	(0.48–4.09)	1.34	(0.43–4.10)
Masaka		Total	295	248 (84)				
							
	Sex	Male	117	97 (83)	0.87	(0.46–1.63)	0.79	(0.40–1.57)
		Female	178	151 (85)	1.00		1.00	
	Age at survey	15–24	30	23 (77)	0.63	(0.23–1.72)	0.60	(0.19–1.94)
		25–34	105	88 (84)	1.00		1.00	
		35–44	106	91 (86)	1.17	(0.55–2.50)	1.21	(0.55–2.64)
		45+	54	46 (85)	1.11	(0.44–2.78)	1.22	(0.47–3.20)
	Education*	None	34	27 (79)	0.92	(0.35–2.40)	0.86	(0.32–2.29)
		Primary 1–4	61	52 (85)	1.38	(0.59–3.23)	1.42	(0.60–3.38)
		Primary 5–7	114	92 (81)	1.00		1.00	
		Secondary +	54	48 (89)	1.91	(0.72–5.08)	1.97	(0.73–5.31)

VCT, voluntary counselling and testing.

* Self-reported for Manicaland, clinic data for Kisesa & Masaka, and combined self-report and clinic data for Karonga.

† Adjusted for all variables.

There was no evidence of interaction between the socio-demographic variables and screening in any site. In Karonga, age at diagnosis remained the only factor associated with screening after controlling for sex, residence and education. In Kisesa, there was borderline evidence that those aged 45 years and over had 2.6 times the adjusted odds of being screened as those aged 25–34 years (*P* = 0.10). In Masaka, there was no evidence of an association with screening uptake for age, education or sex in the adjusted model.

## Discussion

Overall, this study shows substantial variation in HIV service coverage in the four sites. In particular, among adults who had tested HIV-positive in a serological survey, the proportion who knew their status ranged from 25% in Manicaland to 95% in Karonga, indicating that home-based VCT services, such as the one accompanying the HIV surveillance activities in Karonga, are a more effective way of achieving universal diagnosis than are more traditional VCT clinics, such as those provided during survey rounds in Manicaland and Kisesa. While differences in survey dates and population characteristics including HIV prevalence will also partially explain the differences in testing uptake, they nevertheless add weight to arguments that door-to-door testing may be the best way to achieve higher rates of HIV diagnoses, although these services are likely to be relatively expensive.

Nevertheless, this study also showed that higher diagnosis rates do not necessarily translate into better ART coverage, even when HIV clinics are locally available. The highest level of estimated ART coverage was observed in Masaka, despite having a lower proportion of HIV-infected persons who knew their status (56%) than in Karonga (95%). In addition, the probability of ART screening within 2 years of a diagnosis was substantially higher in Masaka (84%) than in Karonga (38%), suggesting that clients who have to actively seek out their test results may be more motivated, or able, to access HIV care. Furthermore, door-to-door testing services that increase the proportion of individuals who are aware of their status need to be balanced by efforts to promote subsequent access to HIV clinics for monitoring and timely treatment initiation, including effective referral systems that include transportation allowances and additional supportive counselling (Nsigaye *et al.* 2009).

Estimated ART coverage was consistently lower among men than women in all sites and tended to be lower among those with least education and those in the youngest age groups (with the exception of Kisesa, where numbers are small). These emerging inequities suggest greater efforts including community mobilisation, tailored health information and youth-friendly initiatives are needed to attract these under-served groups so that they can equally benefit from these life-extending drugs. The particularly low estimated ART coverage in Zimbabwe and Tanzania is worrying, partly reflecting the later introduction of free treatment programmes and decentralisation to rural areas, but also being a function of the shorter interval between ART introduction and the selected survey round, compared with Karonga and Masaka.

There are several limitations to the analysis. Firstly, each site used different methods to ascertain the number of persons who had reached different stages of accessing ART, which may partially account for the differences observed. In Karonga, Kisesa and Masaka, VCT and ART clinic data were linked to cohort data to obtain utilisation rates, with additional reported information on VCT use available from survey questionnaires and on ART initiation for Karonga. In Karonga and Masaka where ART is provided through a research clinic, the linkage procedures use unique identifiers and are likely to effectively capture around 90% of individuals accessing these services. In Kisesa, underestimation of HIV service use is slightly more likely because individuals accessing VCT services outside of the sero-survey were linked to the dataset by counsellors using name, residence, age and sex information, with subsequent referral and ART clinic data linked in using facility-allocated unique identifiers (Nsigaye *et al.* 2009). In Manicaland, VCT and ART use were determined by questionnaire alone, and so underestimation of coverage is also possible, because of a greater opportunity for social desirability bias.

Despite these limitations, this analysis is the first to provide a detailed insight into the degree of access to ART among all HIV-infected persons as well as among those estimated to need treatment, by socio-demographic characteristics in different settings, thus highlighting emerging inequalities in treatment access. Furthermore, these results provide an important baseline for future analyses that monitor trends in HIV service coverage in these settings and provide some evidence of the advantages and disadvantages of different HIV testing and ART provision models that have been debated in recent years in terms of promoting access to ART.
